# Effects and health risk assessments of different spray disinfectants on microbial aerosols in chicken houses

**DOI:** 10.1016/j.psj.2025.105083

**Published:** 2025-03-21

**Authors:** Huaxuan Zhao, Shangmin Li, Junhua Pu, Hongzhi Wang, Huiyong Zhang, Guohui Li, Liang Qu, Xinhong Dou

**Affiliations:** Jiangsu Institute of Poultry Sciences, Yangzhou 225125, PR China

**Keywords:** Disinfectants, Microbial aerosols, Pathogens, Health risk

## Abstract

This study aims to explore the effects of spray processes with four different disinfectants on airborne culturable and pathogenic microbial concentrations, microbial community compositions and health risk assessments in chicken houses. Results indicate that compared to the microbial concentrations before spraying, hypochlorous acid, glutaraldehyde-decamethonium bromide and sodium dichloroisocyanate increase culturable bacteria, culturable fungi, airborne *Staphylococcus* and *Candida albicans*, respectively. Beyond that, the spray processes with different disinfectants have no significant effects on the microbial concentrations. The total relative abundances (RAs) of the screened out 46 pathogenic bacterial genera decrease after spraying with povidone iodine, while increase after spraying with the other three disinfectants, which is opposite for the 35 pathogenic fungal genera. The core bacterial or fungal genera principally interrelate with each other through cooperation. Ammonia (NH_3_) concentrations, relative humidity (RH) and temperature (T) influence bacterial communities in aerosols; while fungal communities are mainly affected by T, particulate matters and nitrous oxide (N_2_O) concentrations. Long-term exposure to aerosols in chicken houses have potential adverse effects on human health and the spray processes with different disinfectants exacerbate the health risks of aerosols via inhalation. Hence, different spray disinfectants cannot significantly reduce the microbial aerosols in real chicken farm environments and the cleaning procedures should be comprehensively reviewed and optimized in livestock and poultry farms.

## Introduction

Aerosols are defined as suspended particles in the air, and microbial aerosols are those with a biological origin (Ruiz-Gil et al., 2020). Livestock and poultry farms are the well-known sources of microbial aerosols, which consist of a variety of biological components, such as bacteria, fungi, pollen, viruses, as well as their cellular fragments and metabolites (Zhao et al., 2023; Zhang et al., 2023). Some of microbial aerosols are pathogens, which may cause allergies and respiratory diseases, posing potential adverse effects to human health (Schultz et al., 2019; Chen et al., 2024). In general, microorganisms discharged from livestock and poultry farms attach to particulate matters and migrate with wind, thus spreading in and out of the breeding houses (Zhou et al., 2021; Zhao et al., 2023).

To prevent epidemic diseases and cut off their transmission paths, breeders will spray the breeding houses with disinfectants, which are expected to kill pathogenic microorganisms (Arabi et al., 2024; Li et al., 2022). In recent years, the frequent occurrences of epidemic diseases in livestock and poultry farms have intensified the widespread use of disinfectants (Li et al., 2022). Common disinfectants include electrolyzed water, hypochlorous acid, hypochlorite, glutaraldehyde, quaternary ammonium salts, povidone iodine, etc. (Rahman et al., 2023; Tong et al., 2024). Spraying 120 ml/m^2^ slight acidic electrolyzed water reduced the airborne bacterial concentrations by 0.7 ∼ 0.37 log10 CFU/m^3^ in a laying hen house (Zheng et al., 2013). Spraying with 80 ml/m^2^ slight acidic electrolyzed water reduced the culturable bacteria (<2.1 μm) by up to 49 % in aviary hen houses (Zheng et al., 2014). The disinfection process with sodium dichlorocyanurate by thermonebullization for 65 minutes significantly decreased the eggshell microbial load by over 1 log10 CFU per egg (Keïta et al., 2016). Spraying with chlorous acid water or NaOCl could effectively reduce *C. jejuni* and spoilage bacteria on chicken skin (Vetchapitak et al., 2021). The combination of quaternary ammonia and glutaraldehyde partially and completely inactivated the infectious bursal disease virus and avian influenza viruses, respectively (Figueroa et al., 2017). However, all disinfectants are not effective and efficient and the actual effects of disinfectants are doubtful in the practice of breeding and production subject to the severe organic pollutants, opening breeding houses and properties of disinfectants, etc. (Marin et al., 2009; Stringfellow et al., 2009). Ineffective disinfection procedures can increase the microbial contaminations and facilitate the spread of microorganisms (Morgan et al., 2022). Additionally, although these spray disinfectants are low toxicity, the prolonged exposure to disinfectants may lead to the development of drug resistance of microorganisms in breeding houses and finally reduce the killing effect of disinfectants on pathogenic microorganisms (Beier et al., 2021; Fernandes et al., 2024; Mpongwana et al., 2024). Hence, the influences of different disinfectants on airborne culturable microbial concentrations and microbial community compositions deserve further study.

The spray processes alter the environmental parameters of the breeding houses and affect the microbial compositions in aerosols in turn. The spray processes can reduce concentrations of particulate matters and then reduce the numbers of microorganisms attached to the surface of particulate matters (Zhao et al., 2023). However, the spray processes may also increase moisture contents and reduce the temperature in breeding houses, which probably increase some microorganism activities and promote the proliferation and expansion of these microorganisms (Islam et al., 2019; Zhong et al., 2016). At present, the key environmental factors that affect microbial community compositions in breeding houses are still not clear and need further investigation.

Quantitative microbial risk assessment (QMRA) recommended by the USEPA has been used to evaluate exposure risks of microbial aerosols. There have been some understandings of the health risk assessments of bacterial aerosols in municipal landfill (Liang et al., 2023), rotavirus and norovirus emitted from water spray park (Pasalari et al., 2022), *Staphylococcus aureus* in wastewater treatment plants (Yan et al., 2021), bioaerosols in an industrial park and the adjacent houses (Zhang et al., 2023). However, the impacts of spray processes with different disinfectants on the health risks of microbial aerosols in breeding houses have not been reported.

This study aimed to explore the effects of spray processes with different disinfectants on microbial aerosols in chicken houses and to assess the health risks of microbial aerosols. Therefore, four chicken houses were selected to investigate the influences of different spray disinfectants on the concentrations of airborne culturable or pathogenic microorganisms in chicken houses. High-throughput sequencing technology was used to explore the airborne microbial community composition changes. The pathogenic and the core microorganisms in aerosols of chicken houses were screened out. The influences and contributions of environmental factors to the airborne microbial communities were explored and the health risks of culturable microbes were evaluated. The results are expected to provide data support for the control of microbial aerosols pollution, the screening of spray disinfectants and the protection of breeders in chicken houses.

## Materials and methods

### Basic information of study sites

This study was conducted in July 2024 at the experimental base of Jiangsu Institute of Poultry Science located in Yangzhou, China. Four chicken houses with the same layouts and equipped with high-pressure atomizers were selected for the experiment. The high-pressure atomizers (DCW-3-4) were purchased from Shandong Dechang Environmental Technology Co., Ltd with a spray speed of 21 L/min and the particle sizes of droplets were controlled at 80 ∼ 120 μm.

### Experimental design

The four chicken houses (named S1, S2, S3, S4) were sprayed with four disinfectants, including hypochlorous acid, glutaraldehyde-decamethonium bromide, povidone iodine and sodium dichloroisocyanate, respectively. Hypochlorous acid disinfectant was purchased from Nantong Flash Water Biotechnology Co., Ltd. The effective chlorine content was 1500 ± 225 mg/L and the spray concentration was 5 %. Glutaraldehyde-decamethonium bromide disinfectant with a concentration of 50 g/L was obtained from Huiqianfang Biotechnology Co., Ltd and the spray concentration was 0.05 %. Povidone iodine disinfectant with a concentration of 100 g/L was purchased from Zhenjiang Victor Pharmaceutical Co., Ltd and the spray concentration was 0.1 %. Sodium dichloroisocyanate disinfectant was obtained from Shanghai Qipiao Animal Health Products Co., Ltd. The effective chlorine content was 10 % and the spray concentration was 0.1 %. The four chicken houses were sprayed with different disinfectants at 9:00 am every Monday, Wednesday and Friday. The spray time was about 1 minute and the total volume of each disinfectant solution was about 20 L.

Culturable microbes and typical pathogenic microbes were collected with the six-stage cascade impactor sampler (FA-1, Changzhou Poussen Electronic Instrument Factory). Before sampling, all instruments and tools were sterilized with 75 % ethanol. The culturable bacteria, culturable fungi, pathogenic bacteria (*Salmonella, Escherichia coli* and *Staphylococcus*) and pathogenic fungi (*Candida albicans*) were collected on the nutrient agar plates (with 5 mg/L nystatin to inhibit the growth of fungi), Sabouraud glucose agar plates (with 4 % chloromycetin to inhibit the growth of bacteria), Xylolysine deoxycholic acid agar (XLD) plates, McConkey agar plates, *Staphylococcus* selective agar plates and *Candida* chromogenic plates at a flow rate of 28.3 L/min for 1 minute in the absence of spray disinfection and within 30 minutes after the spray disinfections, respectively. In addition, an air sampling instrument (Laoying 2030D) was used to collect TSP samples as the microbial aerosols at the flow rate of 16.7 L/min for 18 h on the polycarbonate filter membranes (φ47 mm, 0.45 μm) before and after spraying. The polycarbonate filter membranes were autoclave sterilized (120°C, 20 min) in advance. At the end of sampling, the membranes were placed horizontally in the filter boxes. Both the six-stage cascade impactor sampler and the air sampling instrument were at the sampling height of 1.5 m. Each treatment was repeated three times and the aerosol samples of each treatment were mixed into one sample. The collected samples before and after the spray processes with hypochlorous acid, glutaraldehyde-decamethonium bromide, povidone iodine and sodium dichloroisocyanate were numbered as CK1, P1, CK2, P2, CK3, P3, CK4 and P4. After sampling, the plates and membranes were stored with ice packs and transferred back to the laboratory as quickly as possible. The *Salmonella* and *Escherichia coli* plates were incubated at 37°C for 18 ∼ 24 h; the culturable bacteria and *Staphylococcus* plates were incubated at 37°C for 48 h; the culturable fungi and *Candida albicans* plates were incubated at 25°C for 72 h.

The concentrations (CFU/m^3^) and the removal efficiencies of culturable and pathogenic microbes were calculated using the following (1), (2).

(1) $$\text{Concentration}\;=\;\frac{\text{TC}\times 1000}{T\times 28.3}$$

(2) $$\text{Removal}\;\text{efficiency}\;=\;\frac{C_{\text{before}}-C_{\text{after}}}{C_{\text{before}}}\times 100\%$$

Where TC is the total colony (CFU), T is the sample time (min), C_before_ is the concentration before spraying, C_after_ is the concentration after spraying.

### Detections of environmental parameters

The temperature (T), relative humidity (RH), wind speed (WS), ammonia (NH_3_) concentration, nitrous oxide (N_2_O) concentration, particulate matters (TSP, PM_10_ and PM_2.5_) concentrations of the chicken houses during the experiment were also measured. The T and RH were measured by a thermohygrometer (HP31, Rotronic). The measurement accuracy and resolution are ± 0.3°C and 0.01°C for T and ± 2 % and 0.01 % for RH. The WS was measured by a hot-wire anemometer (LB-FS80, Lubo). The measurement accuracy and resolution are ±3 % and 0.01 m/s. The NH_3_ and N_2_O concentrations were detected by a portable multicomponent gas analyzer (JK60-VI, Shenzhen Jishunan Technology Co. Ltd). The measurement ranges and resolutions are 0 ∼ 100 ppm and 0.01 ppm for NH_3_ and 0 ∼ 1000 ppm and 0.1 ppm for N_2_O. The volume concentration unit (ppm) of NH_3_ and N_2_O were converted to mass concentration unit (mg/m^3^) when analyzing the data. A particulate matter monitor (DUSTTRAK II 8530, TSI) was used to measure the concentrations of TSP, PM_10_ and PM_2.5_. It can take 10 measurements in 1 min and automatically record the average value. The measurement range of the particulate matter monitor is 0.001 ∼ 400 mg/m^3^ with a resolution of ± 0.1 % or 0.001 mg/m^3^, whichever is the greater.

### DNA extraction, PCR amplification and sequencing analysis

The collected membranes were used to extract DNA with soil genomic DNA extraction kit (Sangon Biotech, Shanghai, China). The extracted DNA, primers target for V3-V4 region of bacterial 16S rDNA (341F: 5’-CCTACGGGNGGCWGCAG -3’, 806R: 5’-GGACTACHVGGGTATCTAAT-3’) or for the Internal Transcribed Spacer (ITS) region of fungi (ITS1F: 5’-CTTGGTACTTTAGAGGAAGTAA-3’, ITS2R: 5’-GCTGCGTTCTTCATCGATGC-3’) were used for PCR amplification. The PCR products were recovered through agarose gel, then purified using the AxyPrep DNA Gel Extraction Kit (Axygen Biosciences, Union City, CA, USA). The successful PCR products were sequenced using the Illumina MisSeq PE300 sequencer (Gene Denovo Biotechnology, Guangzhou, China). Following the consolidation of the original data, the bacterial and fungal gene sequences were chosen through quality filtration and evaluation using QIIME (v1.9.1, http://qiime.org/), with a 97 % identity threshold set. The species annotation of bacteria and fungi was performed based on the Silva 138 and Unite 8.0 database, respectively.

### Screening of human pathogens

The human pathogens in aerosols of chicken houses were screened out according to the list of human pathogenic bacteria and fungi (Table S1) constructed by Guo et al. (2021) based on the Ministry of Health of the People's Republic of China and some references (Black, 2012; Ijaz et al., 2016; Fan et al., 2019; Liang et al., 2020; Limon et al., 2017; Qi et al., 2020).

### Health risk assessment

The exposure risk to culturable microbes through inhalation was assessed according to the model recommended by the USEPA. The average daily dose rates (ADD) through inhalation for adult males, adult females, and children and non-cancer risk for inhalation were evaluated (Zhang et al., 2023). The ADD and the hazard quotient (HQ) of non-cancer risk for inhalation was calculated by (3), (4).

(3) $$\text{ADD}\;=\;\frac{C\times \text{IR}\times \text{EF}\times \text{ED}}{\text{BW}\times \text{AT}}$$

(4) $$\text{HQ}\;=\;\frac{\text{ADD}}{\text{RfD}}$$

Where C is the airborne microbial concentration (CFU/m^3^), IR is the inhalation rate (m^3^/d), EF is the exposure frequency (day/year), ED is the exposure duration (year), BW is the bodyweight (kg), AT is the average lifetime (day), RfD is the reference dose (CFU/g·d). The specific parameters are shown in Table S2.

### Statistical analysis

SPSS 19.0 and Origin 9.0 were applied for the basic data analysis. The networks of species correlations were constructed with spearman correlation coefficients. Canonical correlation analysis, variance partitioning canonical correspondence analysis and heatmap analysis were used to illustrated intuitively the relationships between the environmental factors and microbial communities.

## Results and discussion

### Effects of different disinfectants on microbial concentrations and particle size distributions

**Fig. 1:**
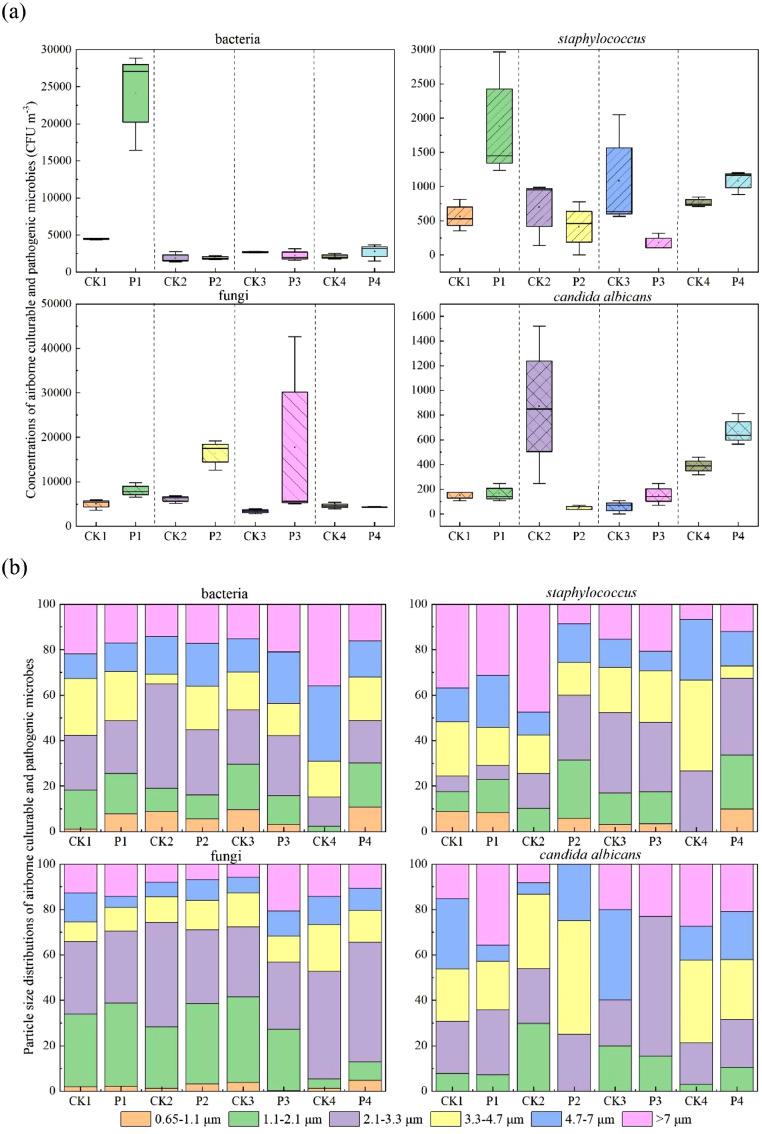
Concentrations (a) and particle size distributions (b) of airborne culturable and pathogenic microbes before and after spraying with different disinfectants.

To understand the effects of different spray disinfectants on microbial aerosols and subsequently assess the human health risks in chicken houses, it is necessary to first test the concentrations of airborne culturable microorganisms. Comparing the airborne culturable bacterial concentrations in chicken houses before and after spraying with hypochlorous acid, glutaraldehyde-decamethonium bromide, povidone iodine and sodium dichloroisocyanate (Fig.1a), it was found that the average airborne bacterial concentrations of the four chicken houses were 1908 ∼ 4487 CFU/m^3^ before the spray processes and 1919 ∼ 24098 CFU/m^3^ after the spray processes. Referring to the Standards for indoor air quality of China (GB/T 18883-2022), the bacterial concentrations were all > 1500 CFU/m^3^, manifesting the negative impact on breeders. Besides, compared to average concentrations of culturable bacteria before spraying, the spray process with hypochlorous acid significantly increased the average concentration of culturable bacteria by 437.01 %. Glutaraldehyde-decamethonium bromide and sodium dichloroisocyanate increased the average concentrations of culturable bacteria by 0.62 % and 32.58 %, while povidone iodine reduced the average concentration of culturable bacteria and the removal efficiency was 17.39 %. Only hypochlorous acid significantly increased the culturable bacteria (P = 0.037), while the last three disinfectants had no significant effect (P ≥ 0.05) on airborne bacterial concentrations in chicken houses before and after spraying, suggesting that spraying with the four disinfectants could not remove the airborne culturable bacteria efficiently.

*Staphylococcus* is a typical pathogenic bacterium. Compared to the average concentrations of *Staphylococcus* before spraying, the spray processes with glutaraldehyde-decamethonium bromide and povidone iodine reduced the average concentrations of *Staphylococcus* with the elimination rates of 40.67 % and 83.70 % (Fig.1a). Hypochlorous acid and sodium dichloroisocyanate increased the average concentrations of airborne *Staphylococcus* by 233.33 % and 41.54 %. Only sodium dichloroisocyanate significantly increased the airborne *Staphylococcus* (P = 0.044), while the other three disinfectants exhibited no significant effect (P ≥ 0.05) on airborne *Staphylococcus* before and after spraying. Therefore, the four disinfectants could not effectively reduce the airborne *Staphylococcus* in chicken houses.

Compared to the average concentrations of culturable fungi before spraying, except that spraying with sodium dichloroisocyanate reduced the average concentrations of airborne culturable fungi with a removal rate of 6.87 %, the spray processes with hypochlorous acid, glutaraldehyde-decamethonium bromide and povidone iodine increased the average culturable fungal concentrations by 60.71 %, 167.95 % and 421.80 % (Fig.1a). Only glutaraldehyde-decamethonium bromide significantly increased the airborne culturable fungi (P=0.007), the other three disinfectants showed no significant difference (P ≥ 0.05) on airborne fungal concentrations before and after spraying. The above results indicated that spraying with the four disinfectants could not eliminate airborne culturable fungi effectively.

*Candida albicans* belongs to the pathogenic fungi commonly found in chicken houses. Compared to the average concentrations of airborne *Candida albicans* before spraying, except that glutaraldehyde-decamethonium bromide showed an inhibitory effect on average concentration of airborne *Candida albicans* with an elimination rate of 94.59 %, spraying with hypochlorous acid, povidone iodine and sodium dichloroisocyanate increased the average concentrations of airborne *candida albicans* by 7.69 %, 160.00 % and 72.73 % (Fig.1a). Only sodium dichloroisocyanate significantly increased the airborne *Candida albicans* (P = 0.028), the other three disinfectants showed no significant difference (P ≥ 0.05) on airborne *Candida albicans* before and after spraying. Hence, the four disinfectants could not efficiently eliminate the airborne *Candida albicans*.

The concentrations of *Salmonella* and *Escherichia coli* before and after spraying with different disinfectants were also investigated. No airborne *Salmonella* and *Escherichia coli* were found in chicken houses, demonstrating that there was no *salmonella* and *Escherichia coli* infection in the four chicken houses during the experiment periods, or *Salmonella* and *Escherichia coli* did not migrate from chickens and manure to aerosols.

Chicken feathers, manure, shed dander, cough sputum, feed and so on will emit abundant microorganisms into the air. The hot and humid summer climate is suitable for the vigorous growth and outbreak of bacteria and fungi (Romero et al., 2022; Guo et al., 2021). The above results showed that the spray processes with different disinfectants could not efficiently remove the airborne microorganisms in aerosols of chicken houses. Hypochlorous acid increased culturable bacteria, glutaraldehyde-decamethonium bromide increased the culturable fungi, sodium dichloroisocyanate increased the airborne *Staphylococcus* and *Candida albicans*. Beyond that, the spray processes with different disinfectants had no significant effects on the concentrations of culturable bacteria, culturable fungi, pathogenic *Staphylococcus* and *Candida albicans*. It can be explained that on the one hand, the severe particulate matter contaminants in chicken houses and relatively low spray concentrations of disinfectants led to the fact that different disinfectants had almost no killing effect on the airborne microorganisms attached to the particulate matters; on the other hand, the spray processes provided appropriate temperature and relative humidity for the growth of some microorganisms, which increased the growth and reproduction of microorganisms (Zhao et al., 2023). It suggested that the commonly used disinfectants could not effectively kill the culturable and pathogenic microbes in aerosols in the real chicken farm environments.

The microbial aerosols with different aerodynamic diameters can deposited at different locations after penetrating the human respiratory system and causing diverse hazards to human body (Chen et al., 2024). As shown in Fig. 1b, the particle size distributions of airborne culturable bacteria and pathogenic *Staphylococcus* were relatively uniform; the culturable fungi were mainly concentrated in 1.1 ∼ 3.3 μm; while there were no *Candida albicans* on the particles (0.65 ∼ 1.1 μm). Microbial aerosols in the inhalable size (< 4.7 μm) can penetrate the bronchi and alveoli into the blood and spread throughout the human body, seriously endangering the human health (Mallik et al., 2020). 30.90 % ∼ 70.28 % of airborne culturable bacterial aerosols and 42.37 % ∼ 74.29 % of pathogenic *Staphylococcus* aerosols were in the inhalable sizes (<4.7 μm), while the proportions of airborne culturable fungal aerosols and pathogenic *Candida albicans* aerosols of inhalable size were 68.10 % ∼ 87.20 % and 40.00 % ∼ 86.49 %. Moreover, Fig. 1b intuitively presented that glutaraldehyde-decamethonium bromide, povidone iodine and sodium dichloroisocyanate increased the proportions of airborne *Staphylococcus, Candida albicans* and culturable bacteria in the inhalable size, respectively. Additionally, the proportions of airborne microbial aerosols in coarse particles (>2.1 μm) were 69.92 % ∼ 97.75 % for culturable bacteria, 66.30 % ∼ 100 % for *Staphylococcus*, 58.48 % ∼ 94.66 % for culturable fungi and 70.27 % ∼ 100 % for *Candida albicans*, demonstrating that these microorganisms were principally concentrated in coarse particles, which was similar to the findings in the outdoor environment (Chen et al., 2024).

### Microbial diversity analysis

The α diversity indices visually showed the richness, diversity and distribution uniformity of microbial communities. For bacterial communities (Table 1), the Chao 1 indices, observed species and ACE indices increased after spraying with all the four disinfectants, manifesting the increased richness of bacterial communities in aerosols. The Shannon indices, Simpson indices and PD whole tree indices increased after the spray processes with hypochlorous acid, glutaraldehyde-decamethonium bromide and povidone iodine, suggesting that the three disinfectants increased the bacterial diversities. However, after spraying with sodium dichloroisocyanate, the Shannon index, Simpson index and PD whole tree index remained basically unchanged, indicating that sodium dichloroisocyanate had little effect on the diversities of bacterial communities in aerosols. The Pielou indices of bacterial communities remained basically stable before and after spraying with the four disinfectants, suggesting that the spray processes only slightly influenced the uniformities of bacterial community distributions.

**Table 1:** α diversity indices of bacterial communities in aerosols of chicken houses

Index	Chao1	Observed species	ACE	shannon	simpson	PD whole tree	Pielou
CK1	2156.16	2102	2231.31	7.97	0.988	309.40	0.722
P1	2802.73	2757	2882.31	8.33	0.989	416.24	0.729
CK2	2129.48	2066	2202.49	8.17	0.987	304.23	0.742
P2	2877.39	2843	2949.37	8.48	0.990	433.41	0.739
CK3	1710.07	1647	1764.80	7.73	0.983	248.48	0.724
P3	2835.05	2797	2915.98	8.29	0.985	429.01	0.724
CK4	1803.18	1743	1864.56	7.85	0.987	259.53	0.729
P4	1882.24	1823	1955.60	7.79	0.986	266.42	0.719

All the α diversity indices of bacterial communities were higher than those of fungal communities, suggesting that the bacterial communities had greater richness, diversity and distribution uniformity than fungal communities in chicken houses (Table 2). For fungal communities, the Chao 1 indices, observed species and ACE indices increased after spraying with hypochlorous acid and glutaraldehyde-decamethonium bromide, suggesting the increased richness of fungal communities; while the three indices decreased after spraying with povidone iodine and sodium dichloroisocyanate, demonstrating that these two disinfectants reduced the richness of fungal communities. After spraying with hypochlorous acid and glutaraldehyde-decamethonium bromide, the Shannon indices, Simpson indices and PD whole tree indices also increased, indicating the increased diversity of fungal communities. In contrast, the spray process with povidone iodine decreased the Shannon, Simpson and PD whole tree indices and the spray process with sodium dichloroisocyanate reduced the PD whole tree index but basically did not alter the Shannon and Simpson indices, demonstrating that the two disinfectants reduced the fungal diversities. The spray processes with hypochlorous acid and povidone iodine decreased the Pielou indices, manifesting the reduction of distribution uniformity of fungal communities; while the Pielou indices increased after spraying with glutaraldehyde-decamethonium bromide and sodium dichloroisocyanate, indicating the enhancement of fungal distribution uniformity.

**Table 2:** α diversity indices of fungal communities in aerosols of chicken houses

Index	Chao1	Observed species	ACE	shannon	simpson	PD whole tree	Pielou
CK1	1081.64	955	1096.81	6.44	0.968	211.61	0.650
P1	1374.09	1175	1400.26	6.52	0.971	245.67	0.639
CK2	1222.74	1037	1229.17	5.87	0.932	215.71	0.586
P2	1581.82	1347	1603.00	6.25	0.962	272.51	0.601
CK3	1212.72	1040	1267.13	5.80	0.937	218.81	0.578
P3	925.50	780	933.33	5.38	0.919	170.08	0.560
CK4	1596.56	1375	1654.56	6.62	0.975	273.87	0.635
P4	1204.79	1033	1206.60	6.63	0.974	222.51	0.662

In conclusion, different spray disinfectants increased bacterial richness and diversity, but had little effect on bacterial distribution uniformity in chicken houses. Hypochlorous acid and glutaraldehyde-decamethonium bromide increased, while povidone iodine and sodium dichloroisocyanate decreased the fungal richness and diversity. Hypochlorous acid and povidone iodine decreased, while glutaraldehyde-decamethonium bromide and sodium dichloroisocyanate increased the fungal distribution uniformity.

Non-metric multi-dimensional scaling (NMDS) analysis had been further used to explore the differences of microbial communities in aerosols of chicken houses before and after spraying with the four disinfectants. Fig. S1a showed that the bacterial aerosol samples before and after spraying with hypochlorous acid, povidone iodine and sodium dichloroisocyanate clustered together without obvious separation, while the spray process with glutaraldehyde-decamethonium bromide obviously influenced the bacterial community structures in aerosols. The stress value of 0 proved the accuracy of the model. Comparatively, fungal aerosol samples before and after spraying with different disinfectants all presented obvious differences (Fig. S1b), suggesting that the spray processes exerted greater influences on fungal community structures.

### Changes of microbial community compositions

The four disinfectants changed the airborne microbial community compositions in chicken houses. At the bacterial phylum level, *Firmicutes, Bacteroidota, Actinobacteriota* and *Proteobacteria* were the top four predominant phyla in aerosols, which accounted for 87.95 % ∼ 93.26 % of the overall bacteria (Fig. S2a). The spray process with hypochlorous acid increased the relative abundances (RAs) of *Firmicutes, Actinobacteriota* and *Bacteroidota*, while decreased the RAs of *Bacteroidota* in aerosols of the chicken houses. Spraying with glutaraldehyde-decamethonium bromide increased the RAs of *Firmicutes, Bacteroidota* and *Proteobacteria*, while decreased the RAs of *Actinobacteriota*. The RAs of *Firmicutes* decreased, while the RAs of *Bacteroidota, Actinobacteriota* and *Proteobacteria* increased after spraying with povidone iodine. The spray process with sodium dichloroisocyanate increased the RAs of *Firmicutes* and *Proteobacteria*, while decreased the RAs of *Bacteroidota* and *Actinobacteria*. Based on the above analysis, it could be seen that different disinfectants showed different elimination effects on bacterial phyla. Hypochlorous acid reduced the RAs of *Bacteroidota*, glutaraldehyde-decamethonium bromide decreased the RAs of *Actinobacteriota*, povidone iodine cut down the RAs of *Firmicutes* and sodium dichloroisocyanate reduced the RAs of *Bacteroidota* and *Actinobacteriota* in aerosols of chicken houses.

At the bacterial genus level (Fig. S2b), the RAs of *Staphylococcus* increased and the RAs of *Bacteroides, Chryseobacterium* and *Pedobacter* decreased after spraying with hypochlorous acid. The spray process with glutaraldehyde-decamethonium bromide decreased the RAs of *Staphylococcus, Pedobacter* and *Corynebacterium*; while increased the RAs of *Bacteroides* and *Acinetobacter*. Spraying with povidone iodine reduced the RAs of *Staphylococcus, Romboutsia* and *Pedobacter*, while increased the RAs of *Rothia* and *Chryseobacterium*. The spray processes with sodium dichloroisocyanate increased the RAs of *Lactobacillus* and *Staphylococcus*, while decreased the RAs of *Chryseobacterium*. To sum up, hypochlorous acid mainly reduced the RAs of *Chryseobacterium* and *Pedobacter*, glutaraldehyde-decamethonium bromide decreased the RAs of *Pedobacter* and *Corynebacterium*, povidone iodine cut off the RAs of *Staphylococcus, Romboutsia* and *Pedobacter*, sodium dichloroisocyanate reduced the RAs of *Chryseobacterium* in aerosols of chicken houses.

At the fungal phylum level, the fungal communities in aerosols of chicken houses primarily belonged to *Basidiomycota* and *Ascomycota*, which take up 99.13 % ∼ 99.69 % of the total fungi (Fig. S2c). The spray processes with hypochlorous acid and povidone iodine decreased the RAs of *Basidiomycota*, while increased the RAs of *Ascomycota*. The opposite was true for the spray processes with glutaraldehyde-decamethonium bromide and sodium dichloroisocyanate.

At the fungal genus level (Fig. S2d), spraying with hypochlorous acid increased the RAs of *Hyphopichia* and *Kodamaea* and decreased the RAs of *Irpex* and *Aspergillus*. The spray processes with glutaraldehyde-decamethonium bromide decreased the RAs of *Hyphopichia, Aspergillus* and *Kodamaea*, while increased the RAs of *Irpex, Schizophyllum* and *Tilletia*. After spraying with povidone iodine, the RAs of *Hyphopichia* increased, while the RAs of *Tilletia* reduced. The spray processes with sodium dichloroisocyanate decreased the RAs of *Hyphopichia, Trametes* and *Aspergillus*, while increased the RAs of *Kodamaea*.

As can be seen from the Venn diagrams of the microbial communities in aerosols of chicken houses (Fig. S3), after spraying with the four disinfectants, the common bacterial genera accounted for 58.82 % ∼ 64.09 % and the unique bacterial genera took up 21.29 %, 22.96 %, 24.95 % and 16.28 % of the overall bacterial genera. Likewise, the common fungal genera took up 59.10 % ∼ 66.52 % and the unique fungal genera accounted for 21.07 %, 22.25 %, 11.87 %, 9.09 % after the spray processes. It demonstrated that the spray processes removed some of the bacteria and fungi from aerosols, but introduced others that might come from sprinkler tanks and pipes. About 60 % of bacterial and fungal genera were always present in aerosols, whether spray or not.

### Screening of human pathogens

**Fig. 2:**
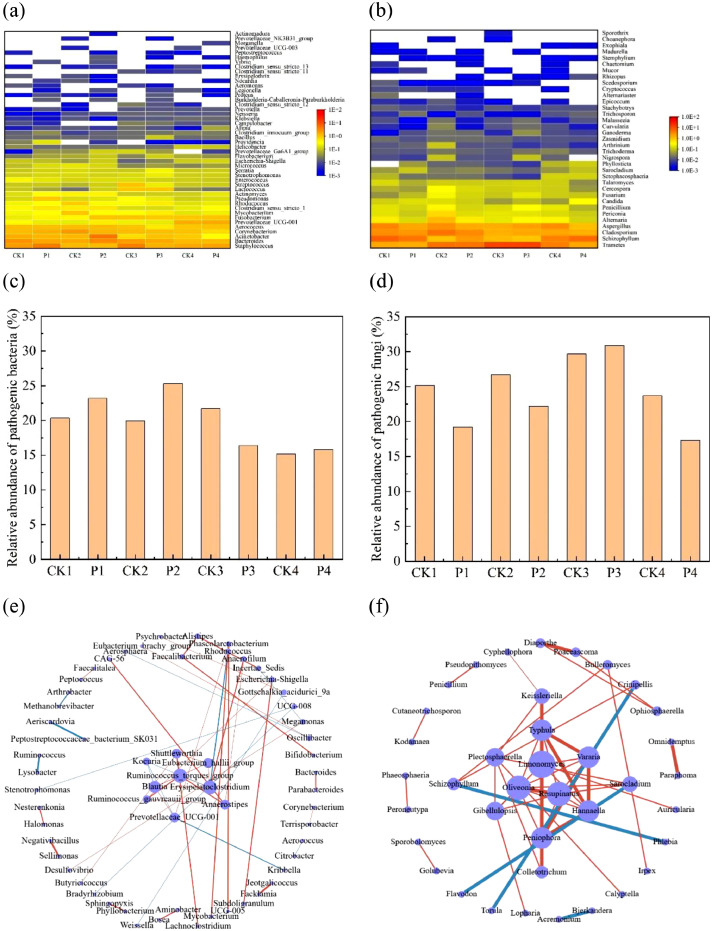
Screening of pathogenic and core microorganisms. Heatmap analysis of the human pathogenic bacteria (a) and fungi (b) at the genus level. Value plot is the relative abundance. The white square means that the pathogen has not been detected. Relative abundances of human pathogenic bacteria (c) and fungi (d). Networks of the core bacterial (e) and fungal (f) genera in aerosols of chicken houses.

Comparing the annotation results of 16S rDNA and ITS sequencing data with the list of human pathogenic bacteria and fungi (Table S1), a total of 46 pathogenic bacterial genera and 35 pathogenic fungal genera were screened out (Fig. 2a, b). The most abundant pathogenic bacteria included *Staphylococcus, Bacteroides, Acinetobacter, Corynebacterium, Aerococcus, Prevotellaceae_UCG-001, Fusobacterium, Mycobacterium, Clostridium_sensu_stricto_1, Rhodococcus, Pseudomonas, Actinomyces, Lactococcus* and *Streptococcus*. Many species of *Staphylococcus* can cause different forms of infections. In particular, *Staphylococcus aureus* easily leads to superficial skin lesions, localized abscesses, food poisoning, toxic shock syndrome and urinary tract infections (Foster, 2002). The RA of *Staphylococcus* in aerosols of chicken houses increased after spraying with hypochlorous acid and sodium dichloroisocyanate, while decreased after spraying with glutaraldehyde-decamethonium bromide and povidone iodine. The changes of pathogenic *Staphylococcus* concentrations in aerosols (Fig. 1a) proved the above result. *Bacteroides* is a human opportunistic pathogen that can cause intra-abdominal infections, postoperative wounds, skin and soft tissue infections (Eitel et al., 2013). Hypochlorous acid and povidone iodine decreased, while glutaraldehyde-decamethonium bromide increased the RAs of *Bacteroides. Acinetobacter*, a kind of human opportunistic pathogenic bacteria and able to adapt to different environments, can cause lung infections, pneumonia, sepsis and bacteraemia for immunocompromised hosts (Dijkshoorn et al., 2007; Ku et al., 2000; Liang et al., 2020; Wilson et al., 2020). Glutaraldehyde-decamethonium bromide and povidone iodine increased, while hypochlorous acid and sodium dichloroisocyanate decreased the RAs of *Acinetobacter*. Certain bacteria in *Corynebacterium*, such as *Corynebacterium diphtheriae* can cause diphtheria and skin infections (Lu et al., 2018). *Mycobacterium* can cause pulmonary disease and is prevalent in water and soil (Croix and Munsiff, 2022; Daley et al., 2022). *Rhodococcus* can cause lung abscesses in immunocompromised patients (Piselli et al., 2022). *Pseudomonas* can cause wound infection and is ubiquitous in the environment (Hasannejad-Bibalan et al., 2021). Some bacterial species of the *Streptococcus* genus can cause childhood pneumonia and meningitis and may be a serious threat to children health (Guo et al., 2021; Mehr and Wood, 2012). *Stenotrophomons* is susceptible to endocarditis and urinary tract infections, which is highly resistant to antibiotics (Tang et al., 2023).

The pathogenic fungal genera in aerosols of chicken houses primarily involved *Trametes, Schizophyllum, Cladosporium, Aspergillus, Alternaria, Penicillium, Candida, Fusarium, Cercospora, Talaromyces, Setophaeosphaeria, Sarocladium, Phyllosticta, Malassezia* and so on. *Cladosporium* can cause subcutaneous and brain abscess (Ibrahim et al., 2022). *Aspergillus*, which produces poisonous aflatoxins, can cause liver damage to humans and animals (Diener et al., 1987; Guo et al., 2021). *Alternaria* can cause allergic respiratory diseases with a high sensitization rate (Abel-Fernández et al., 2023; Chen et al., 2024). *Penicillium* can produce citrinin that damaging the nervous system and kidneys (Zhang et al., 2023). *Candida albicans*, a member of *Candida* genus, can cause gastrointestinal tract, mucosa and skin infections (Limon et al., 2017). Glutaraldehyde-decamethonium bromide decreased, while hypochlorous acid, povidone iodine and sodium dichloroisocyanate increased the RAs of *Candida albicans*, which was mutually confirmed with the result in Fig. 1a.

In general, pathogenic bacterial genera in aerosols of chicken houses accounted for 15.15 % ∼ 25.29 % of the total bacteria; pathogenic fungal genera took up 17.27 % ∼ 30.85 % of the total bacteria (Fig. 2c, d). Hypochlorous acid, glutaraldehyde-decamethonium bromide and sodium dichloroisocyanate increased, while povidone iodine decreased the RAs of total pathogenic bacteria and the opposite was true for pathogenic fungi.

### Determinations of core microorganisms

The airborne microbial communities in chicken houses were dynamic ecosystems. The microorganisms in aerosols were not independent of each other and there were direct or indirect relationships such as competition, cooperation, synergy, antagonism among the microbial species. Network analysis of species correlation is one of the effective means to understand the changes in association patterns among microorganisms. In networks of species correlation, some species are in the hub positions, which play important roles in maintaining the stability of microbial community structures and are called core microorganisms. It is worth investigating the core microorganisms in aerosols of chicken houses, which can provide help for the targeted regulation of the airborne microbes. According to that |spearman coefficient| > 0.5 and P < 0.05, a total of 145 bacterial genera were screened out, which formed 10440 pairs of correlations. Similarly, a total of 101 fungal genera were screened out, which formed 5050 pairs of correlations. Among them, the top 50 pairs of bacterial and fungal correlations were selected to construct the networks and the selected bacterial and fungal genera were defined as core bacteria and fungi (Fig. 2e, f).

59 bacterial genera constructed the top 50 pairs of correlations, of which 14 pairs were negative and 36 pairs were positive. The positive correlations accounted for 72 % of the top 50 pairs of correlations. Moreover, the most critical bacterial genera were *Shuttleworthia, Kocuria, Eubacterium_hallii_group, Ruminococcus_torques_group, Blautia, Erysipelatoclostridium, Ruminococcus_gauvreauii_group, Anaerostipes, Prevotellaceae_UCG-001*, of which the connectivity and abundance were relatively high. Besides, *Prevotellaceae_UCG-001, Bacteroides, Corynebacterium, Aerococcus, Mycobacterium, Rhodococcus, Stenotrophomonas* and *Escherichia-Shigella* belonged to pathogenic bacteria. Among them, *Prevotellaceae_UCG-001* was the core pathogenic bacterial genus. 38 fungal genera constructed the top 50 pairs. Except for the four pairs between *Sarocladium* and *Flavodon, Torula* and *Crinipellis, Acremonium* and *Bjerkandera, Phlebia* and *Schizophyllum*, which were negative correlations, the positive correlations took up 92 % of the top 50 pairs. Furthermore, *Schizophyllum, Sarocladium* and *Penicillium* were pathogenic fungi. Additionally, the most critical fungal genera included *Keissleriella, Typhula, Vararua, Plectosphaerella, Limonomyces, Schizophyllum, Oliveonia, Resupinatus, Sarocladium, Gibellulopsis, Peniophora, Hannaella, Colletotrichum*. Hence, *Schizophyllum* and *Sarocladium* were the core pathogenic fungal genera. Above all, the core bacteria and fungi in aerosols of chicken houses mainly showed cooperative relationships at the genus level. *Prevotellaceae_UCG-001* was the core pathogenic bacterial genus and *Schizophyllum* and *Sarocladium* were the core pathogenic fungal genera.

### Relationships between microbial communities and environmental factors

**Fig. 3:**
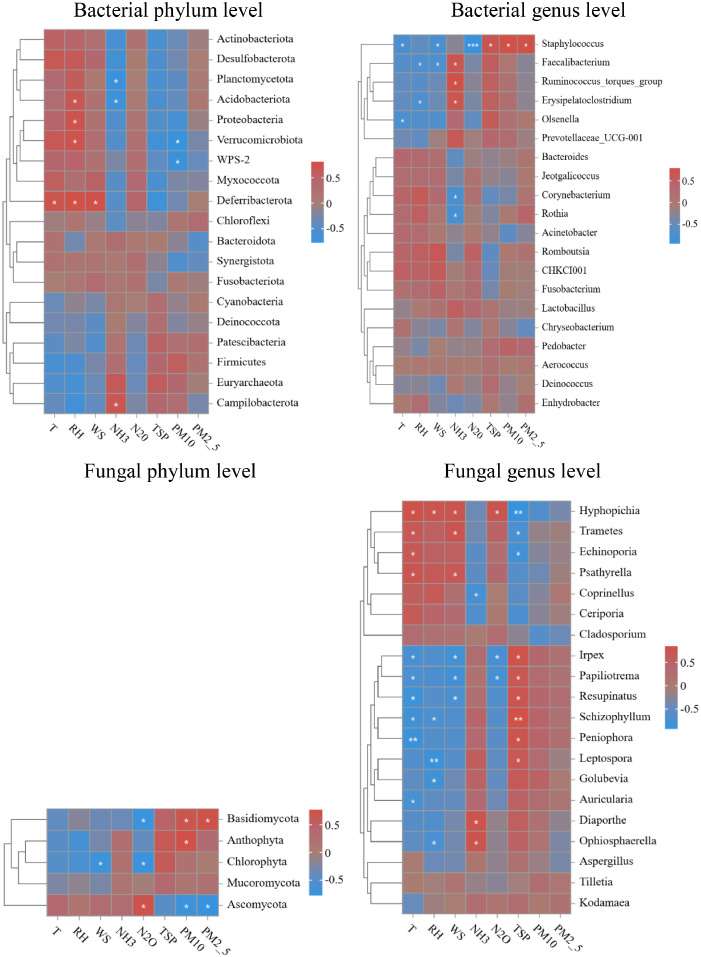
Heatmap analysis of the bacterial or fungal communities and environmental factors at the phylum and genus levels. Differences were assessed by ANOVA and denoted as follows: *** p<0.001, **p<0.01, *p<0.05.

Heatmaps were used to exhibit the spearman correlations between the bacterial or fungal community compositions and environmental factors (Fig. 3). In general, the correlations of microorganisms in aerosols with T, WS, RH and N_2_O concentrations and with NH_3_ and particulate matters (TSP, PM_10_, PM_2.5_) concentrations were just the opposite. For instance, at bacterial phylum level, *Actinobacteriota, Desulfobacterota, Planctomycetota, Acidobacteriota, Proteobacteria, Verrucomicrobiota, WPS-2, Myxococcota* and *Deferribacterota* were positively associated with T, WS, RH and N_2_O concentrations and negatively correlated with NH_3_, TSP, PM_10_ and PM_2.5_ concentrations. At bacterial genus level, *Staphylococcus, Faecalibacterium, Ruminococcus_torques_group, Erysipelatoclostridium, Olsenella* presented negatively correlations with T, WS, RH and N_2_O concentrations and positively associations with NH_3_ and particulate matter concentrations. At fungal phylum level, *Basidiomycota* was positively correlated with PM10 and PM2.5 concentrations, but negatively associated with N_2_O concentrations, while *Ascomycota* was the opposite. At fungal genus level, *Hyphopichia, Trametes, Echinoporia, Psathyrella, Coprinellus* were positively correlated with T, WS, RH and N_2_O concentrations and negatively correlated to NH_3_ and particulate matter concentrations, while *Irpex, Papiliotrema, Resupinatus, Schizophyllum, Peniophora, Leptospora, Golubevia, Auricularia* were negatively correlated with T, WS, RH and N_2_O concentrations and positively associations with NH_3_, TSP, PM_10_ and PM_2.5_ concentrations. This can be explained that the hot and humid environment would promote the productions of a good deal of N_2_O in chicken houses in summer and could be generally alleviated by increasing the wind speed of the fans. The spray processes increased the relative humidity and reduced the concentrations of particulate matters as well as the NH_3_ concentrations, since NH_3_ was dominantly attached to the particulate matters (Zhou et al., 2021).

**Fig. 4:**
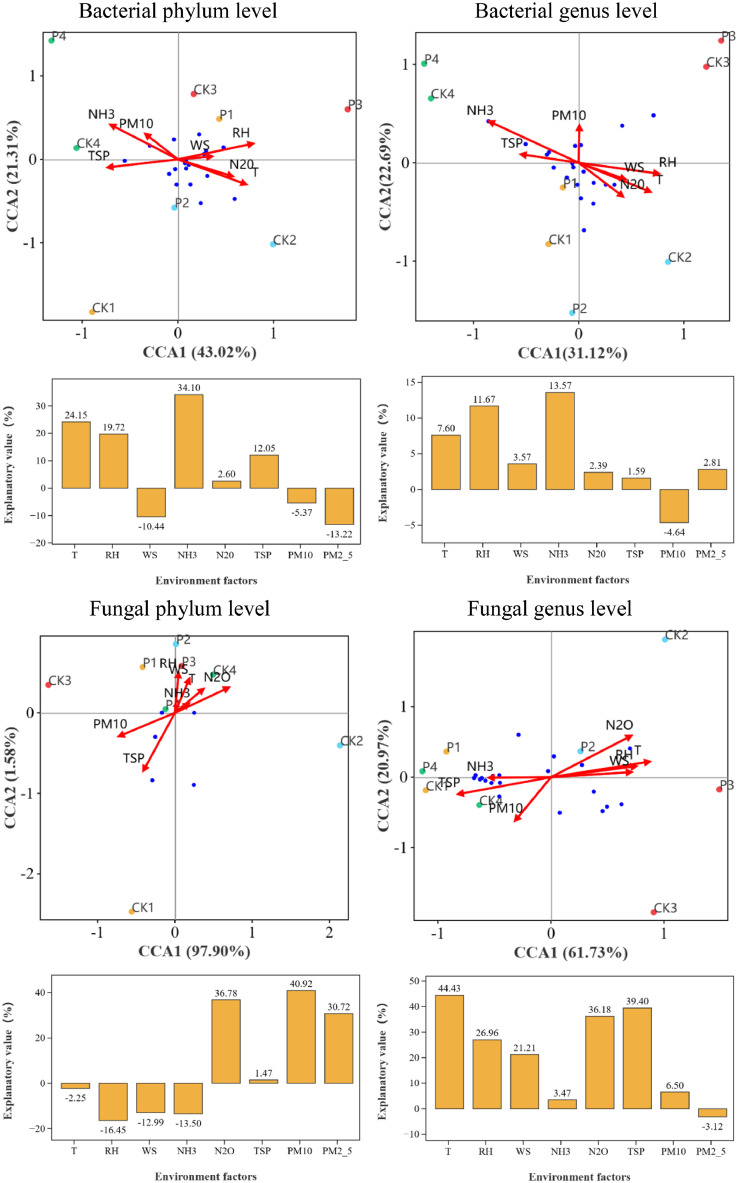
Canonical correlation analysis (CCA) and variance partitioning canonical correspondence analysis (VPA) of bacterial or fungal communities and environmental factors at the phylum and genus levels.

Canonical correspondence analysis (CCA) can intuitively reveal the relationships between aerosol samples, microbial communities and environmental factors in chicken houses (Fig. 4). At the bacterial phylum level, NH_3_ concentration (R^2^ = 0.640, P = 0.088), RH (R^2^ = 0.624, P = 0.094) and T (R^2^ = 0.572, P = 0.12) were relatively strongly associated with the bacterial communities, but none of them reached the significance. At the bacterial genus level, NH_3_ concentration (R^2^ = 0.873, P = 0.048), RH (R^2^ = 0.571, P = 0.137) and T (R^2^ = 0.533, P = 0.181) were associated with the bacterial communities, among which, NH_3_ concentration exhibited a significant correlation with the bacterial communities. Variance partitioning canonical correspondence analysis (VPA) can be used to quantitatively analyze the explanatory degrees of environmental factors to the changes of microbial communities. The results of VPA analysis have intuitively proved that NH_3_ concentration, RH and T were the main environmental factors affecting the bacterial communities and NH_3_ concentration, RH and T explained 34.10 %, 19.72 % and 24.15 % of the changes of bacterial communities at the bacterial phylum level and 13.57 %, 11.67 % and 7.60 % at the bacterial genus level (Fig. 4). Hence, the bacterial communities in aerosols of chicken houses were mainly influenced by NH_3_ concentration, RH and T.

Similarly, at the fungal phylum level, TSP (R^2^ = 0.652, P = 0.084), PM_10_ (R^2^ = 0.576, P = 0.094) and N_2_O (R^2^ = 0.554, P = 0.077) concentrations were strongly correlated with fungal communities, but no significant correlation was reached. At the fungal genus level, there were relatively strong correlations between N_2_O concentration (R^2^ = 0.821, P = 0.015), T (R^2^ = 0.786, P = 0.017), TSP concentration (R^2^ = 0.709, P = 0.046), RH (R^2^ = 0.573, P = 0.117) and fungal communities (Fig. 4). In particular, N_2_O concentration, T and TSP concentration were significantly correlated with fungal communities. Moreover, the VPA analysis demonstrated that at the fungal phylum level, PM_10_, N_2_O and PM_2.5_ concentrations were the three environmental factors dominantly influencing the fungal communities, which explained 40.92 %, 36.78 % and 30.72 % of the changes of fungal communities. At the fungal genus level, T, TSP concentration, N_2_O concentration and RH were the top four environmental factors that showed the greatest impact on fungal community changes, which have explained 44.43 %, 39.40 %, 36.18 % and 26.96 % of the changes of fungal communities (Fig. 4). The above results presented that the fungal communities in aerosols of chicken houses were primarily affected by T, particulate matters and N_2_O concentrations.

### Health risk assessment of airborne culturable microbes

**Fig. 5:**
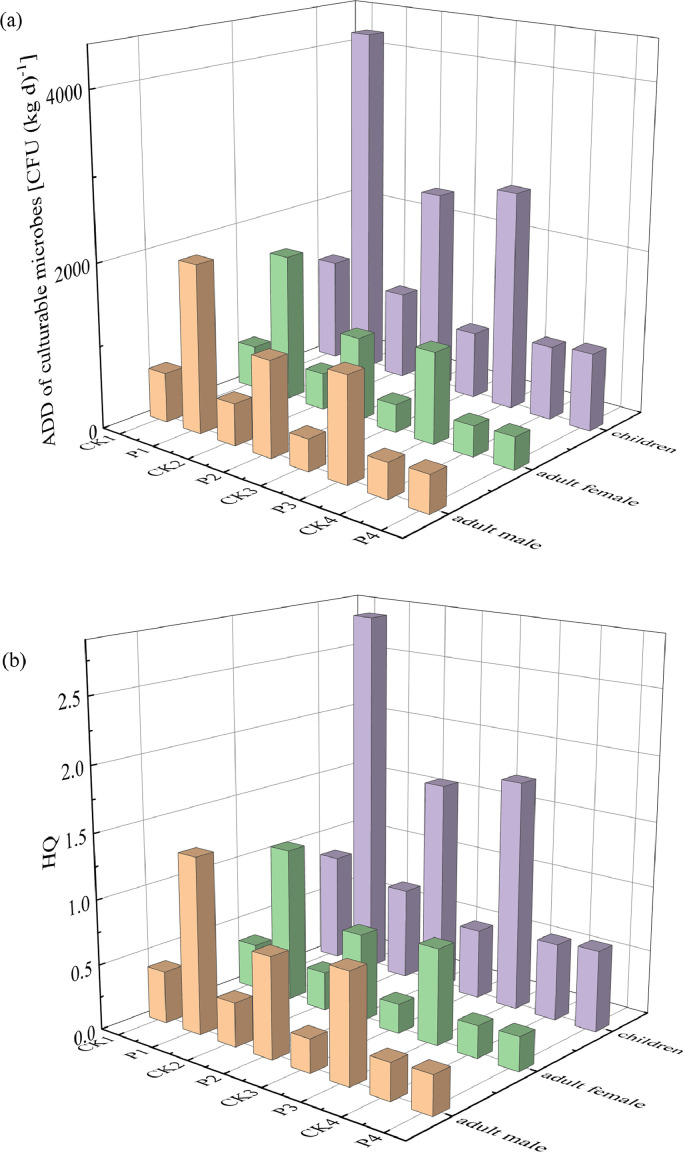
Average ADD (a) and HQ (b) of culturable microbes through inhalation before and after spraying disinfectants.

Long-term exposures to the high concentrations of pathogenic microbial aerosols in chicken houses will cause a variety of chronic diseases and pose serious health risks to human body (Jahne et al., 2015; Zhang et al., 2023). To evaluate the health risks of airborne microbial aerosols, based on the culturable bacteria and fungi concentrations, the average ADD of microbial aerosols for adult male, adult female and children via inhalation were calculated and the values after spraying were greater than those before spraying (Fig. 5a). That is, the spray processes increased the average ADD of microbial aerosols in the chicken houses.

We further assessed the non-carcinogenic risk of microbial aerosols by inhalation and found that the HQ values after spraying were all greater than those before spraying (Fig. 5b). Particularly, the HQ values of microbial aerosols via inhalation for adult male, adult female and children after spraying hypochlorous acid and the HQ values of microbial aerosols via inhalation for children after spraying glutaraldehyde-decamethonium bromide and povidone iodine were all greater than 1, indicating the potential adverse health effects, which was significantly different from the microbial aerosols in residential areas (Chen et al., 2024; Zhang et al., 2023). In summary, the airborne microbial contaminations in chicken houses were far worse than those in residential areas and the wide existence of microbial aerosols in chicken houses would pose potential health risks to the surrounding people. Especially, the spray processes would increase the non-carcinogenic risks of microbial aerosols via inhalation in chicken houses, which might be amplified for vulnerable groups such as children, the elderly, and the pregnant, etc. (Zhang et al., 2023). Given that the disinfectants themselves can pose a threat to gut microbiome and immune function (Xu et al., 2024), it is necessary to re-examine the significance of the spray processes and optimize the cleaning procedures of the livestock and poultry farms.

The above results suggested that the spray processes with four different chemical disinfectants cannot effectively kill culturable and pathogenic microbes in aerosols in real chicken farm environments, but increased the health risks of microbial aerosols via inhalation, which probably attributed to the severe particulate matter contaminants, relatively low spray concentrations and appropriate temperature and relative humidity after spraying. Therefore, in terms of poultry farm management, it is recommended to increase the frequency of manure removal and ground cleaning to reduce particulate matters in chicken houses. Besides, breeders should be equipped with full protective clothing including protective suit, masks, gloves and goggles.

Additionally, this study only investigated the effect of spray processes with four chemical disinfectants on airborne microorganisms in chicken houses. We will explore the killing effects of other types of spray additives, such as plant extracts, microbial agents, enzyme preparations, etc., on the airborne culturable and pathogenic microbes in future research. Moreover, the colonization process of poultry farm microbiota on the surface organs of the breeders such as skin, oral cavity, nasal cavity as well as the interactions between poultry farm microbiota and human microbiota deserve further study.

## Conclusions

The commonly used spray disinfectants cannot effectively kill culturable and pathogenic microbes in aerosols in real chicken farm environments. Hypochlorous acid, glutaraldehyde-decamethonium bromide and sodium dichloroisocyanate increased, while povidone iodine decreased the RAs of total pathogenic bacteria and the opposite was true for pathogenic fungi. The core bacterial and fungal genera mainly exhibited cooperative relationships. The bacterial communities were mainly influenced by NH_3_, RH and T, while T, particulate matters and N_2_O affected the fungal communities in aerosols. Long-term exposure to microbial aerosols in chicken houses caused potential adverse effects and the spray processes with all the four disinfectants increased the health risks via inhalation. Therefore, it is necessary to comprehensively review the roles of spray processes with different disinfectants and optimize the cleaning procedures of the livestock and poultry farms.

## Declaration of interests

The authors declare that they have no known competing financial interests or personal relationships that could have appeared to influence the work reported in this paper.

## Appendix Supplementary materials

Image, application 1
