# Supplementary material for: Effects and health risk assessments of different spray disinfectants on microbial aerosols in chicken houses
Source: Poult Sci. 2025 Mar 21;104(5):105083. doi: 10.1016/j.psj.2025.105083 (PMC11986507; doi:10.1016/j.psj.2025.105083)
Supplement: Supplementary file 1 [file mmc1.docx]

Supplementary data

(a)


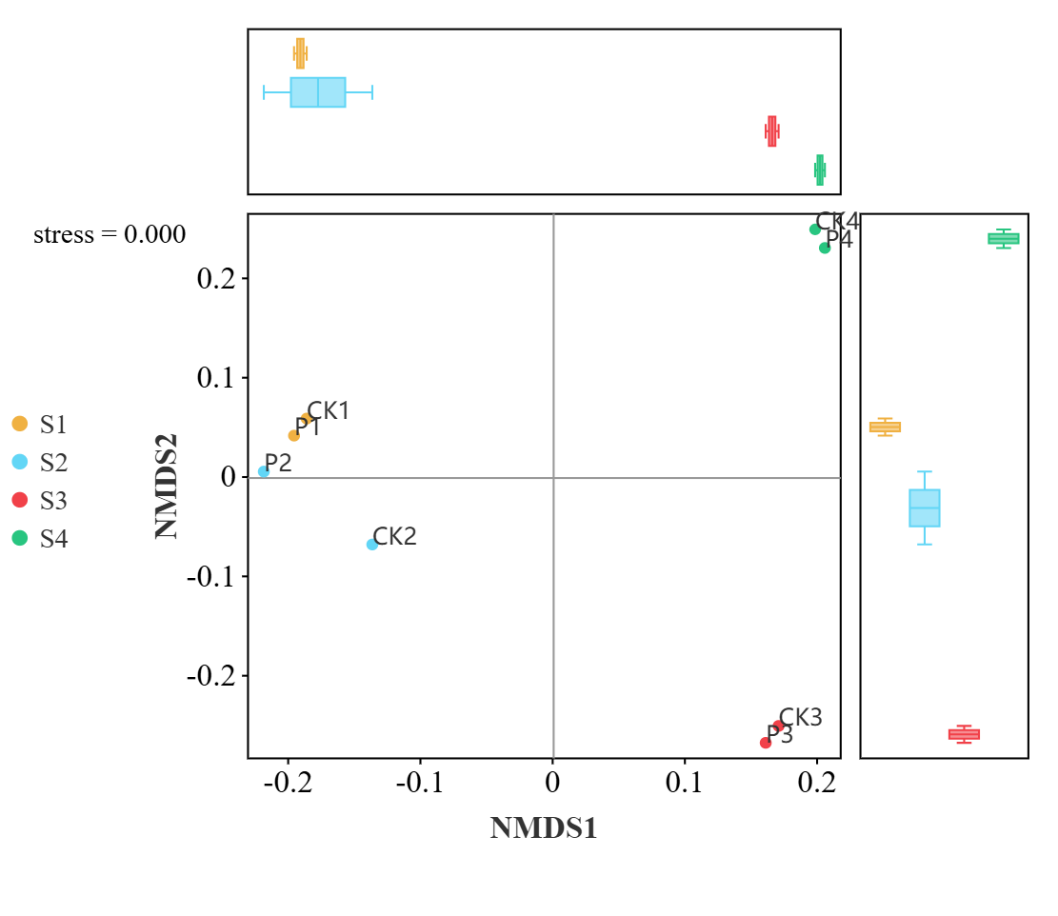


(b)


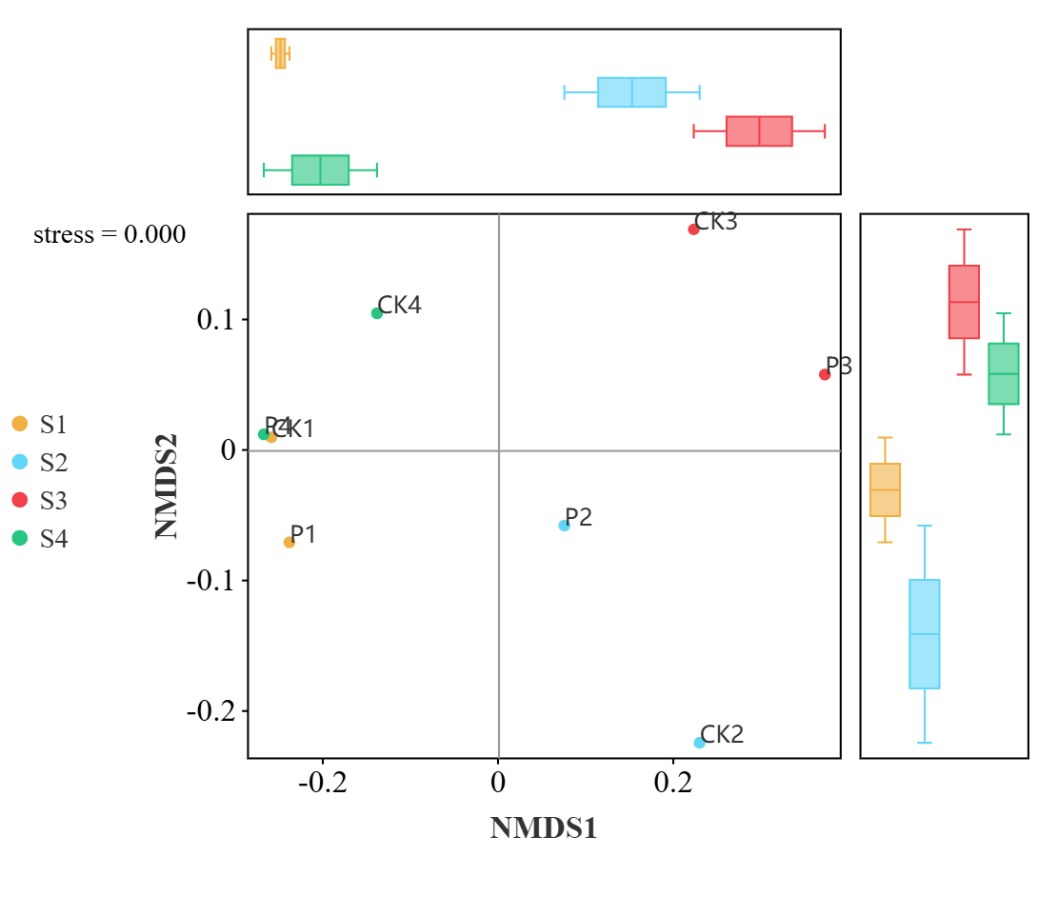


**Fig. S1.** NMDS analysis of microbial communities in aerosols of chicken houses (a: bacterial community; b: fungal community).

(a)


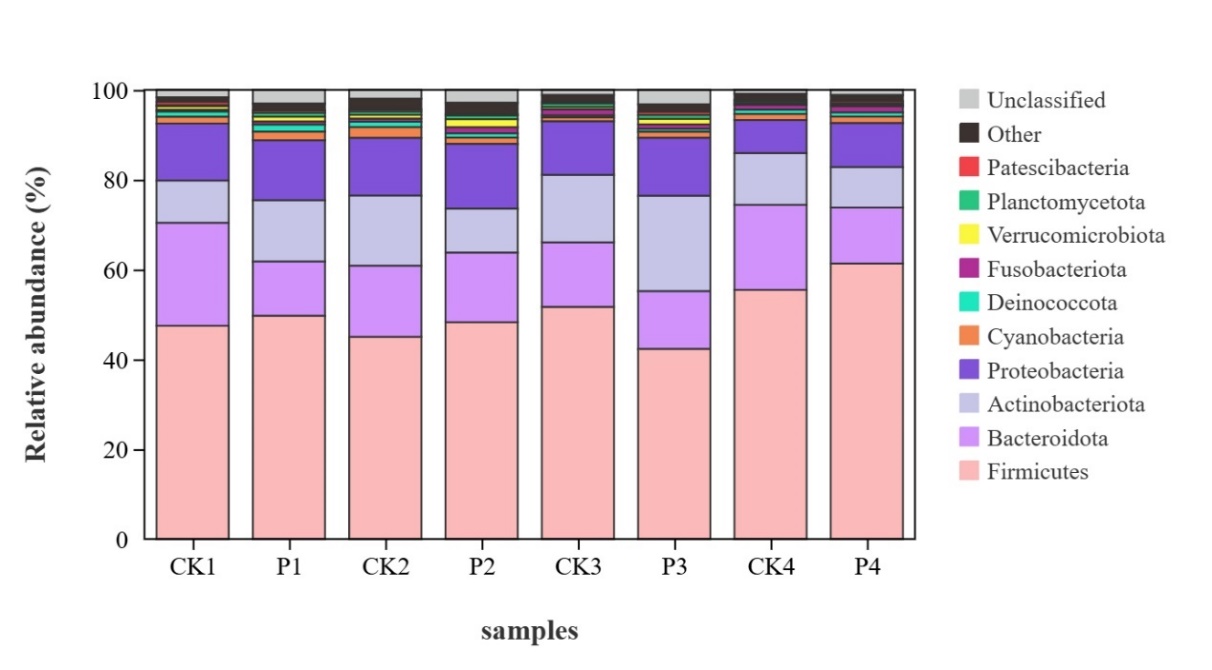


(b)


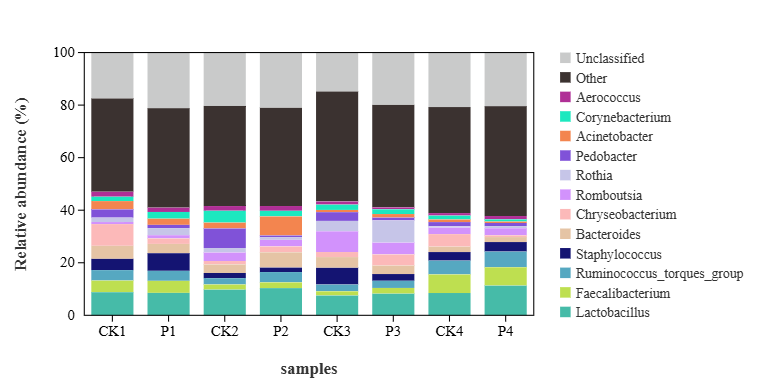


(c)


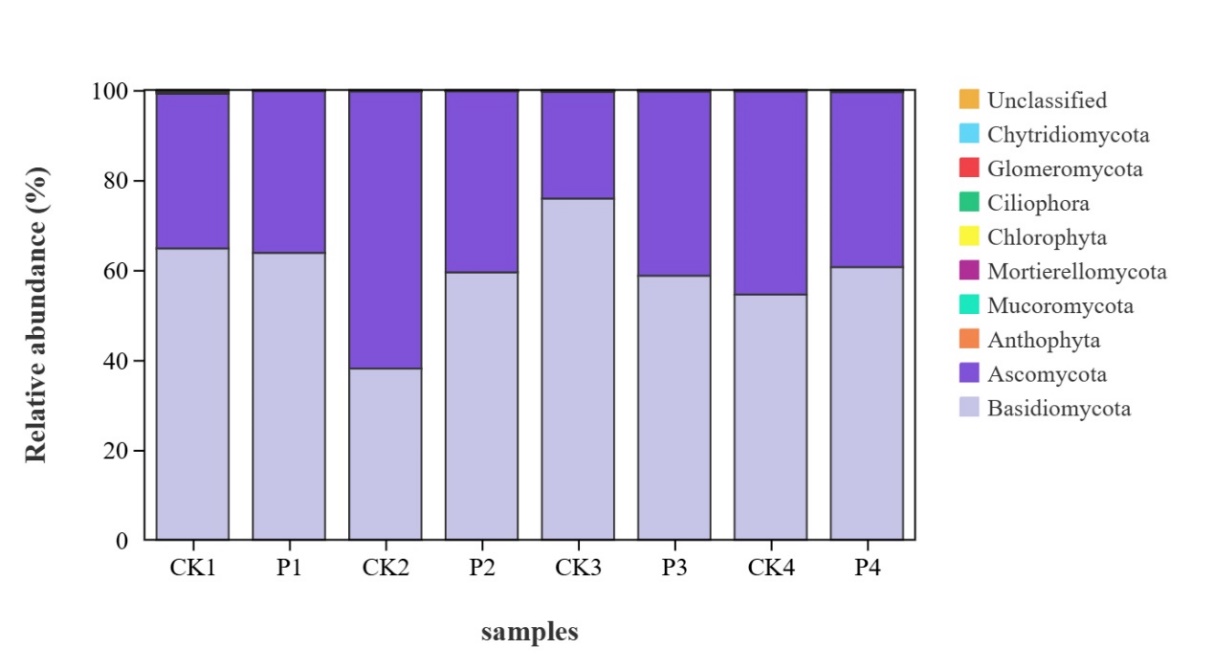


(d)


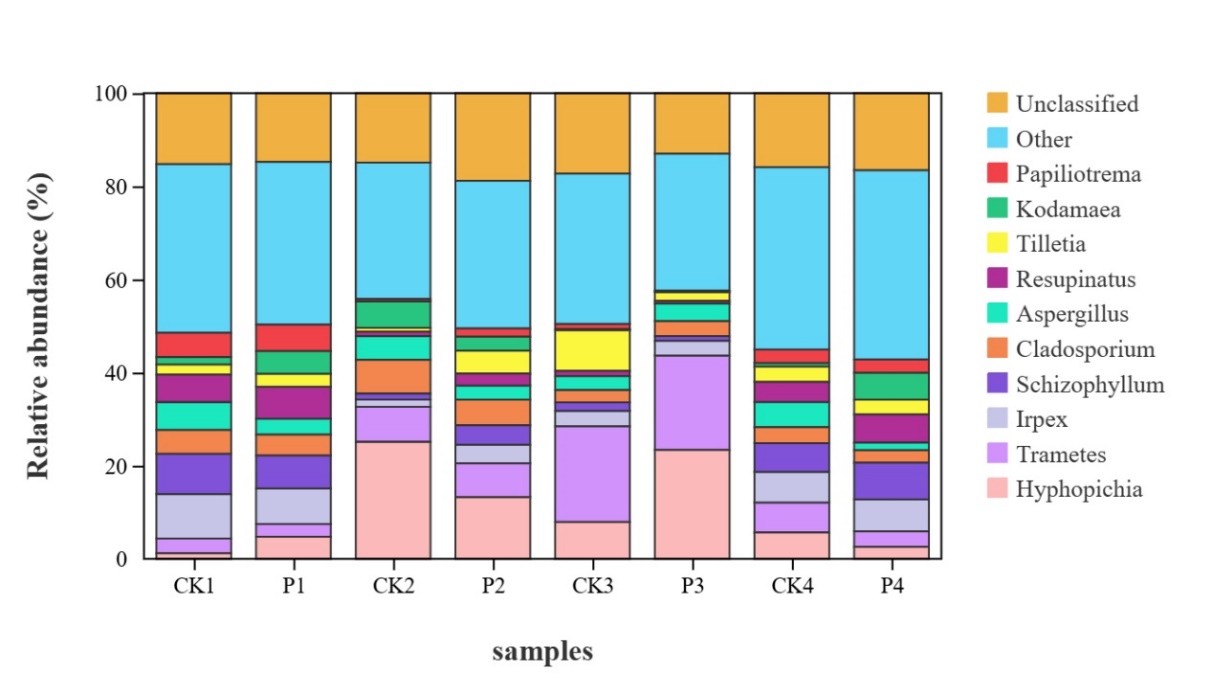


**Fig. S2.** Bacterial (a, b) and fungal (c, d) community compositions in aerosols of chicken houses at the phylum and genus levels.

(a)


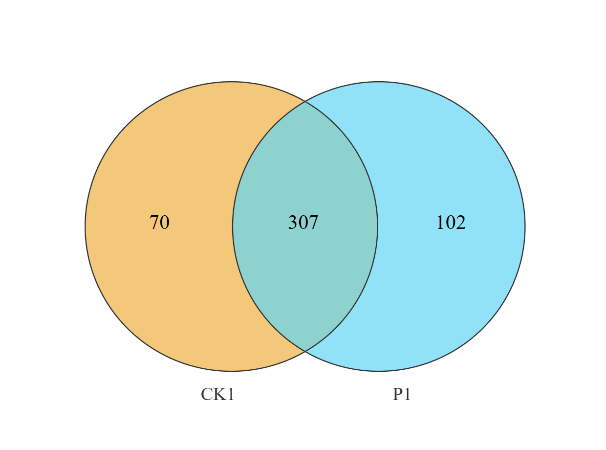

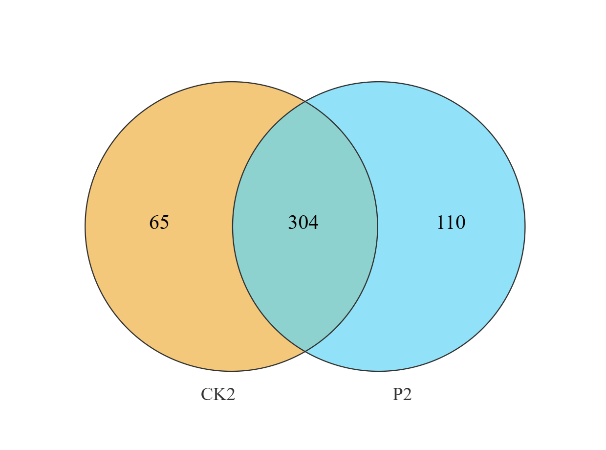


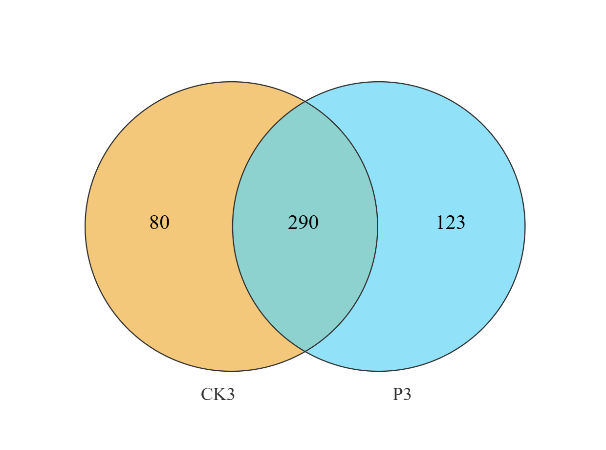

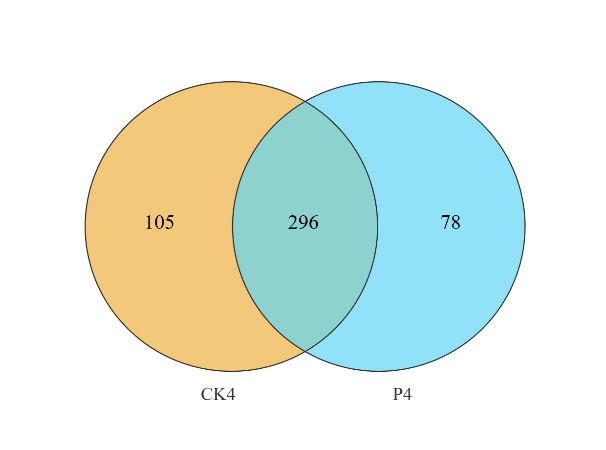


(b)


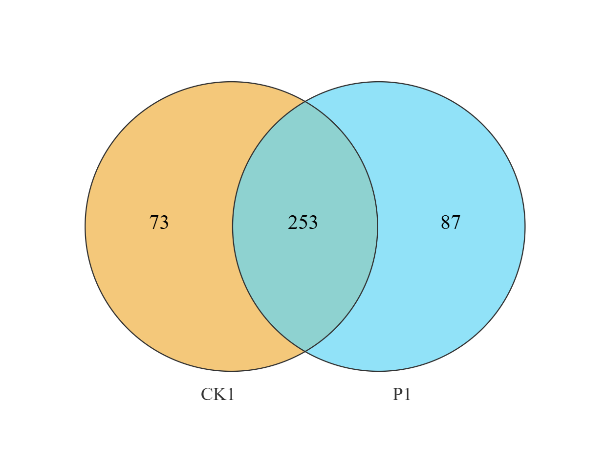

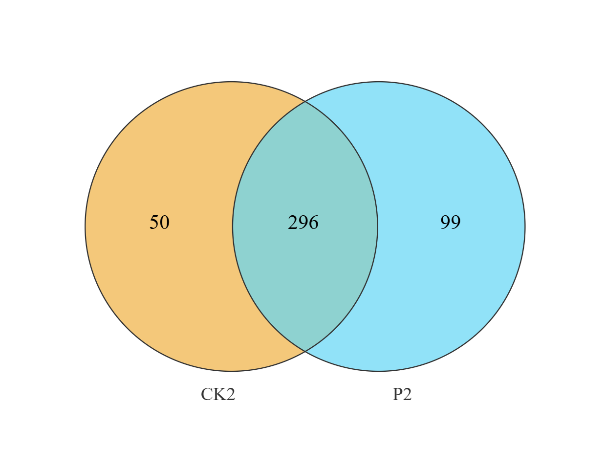


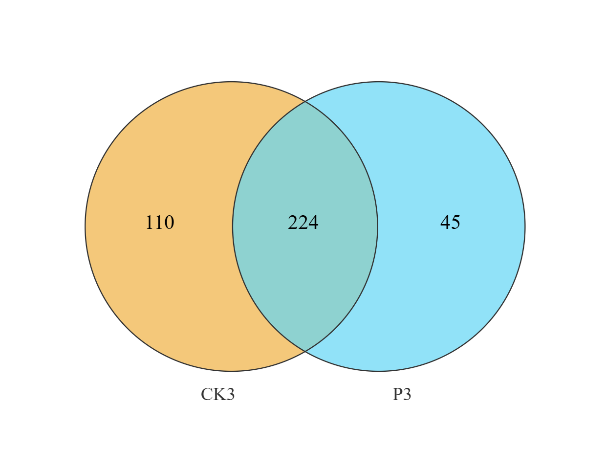

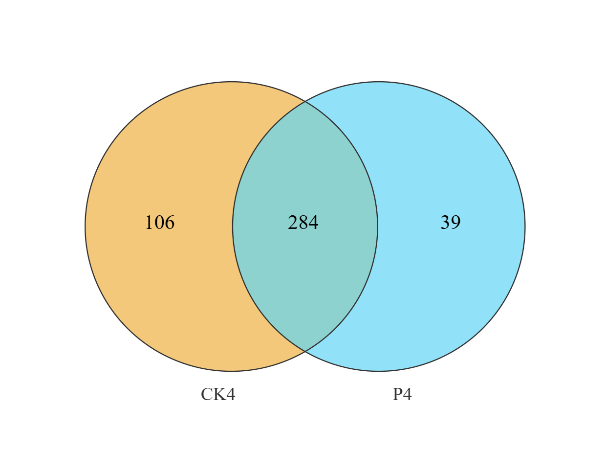


**Fig. S3.** Venn diagram of microbial communities in aerosols of chicken houses (a: bacterial genus level; b: fungal genus level).

**Table S1** List of human pathogenic bacteria and fungi

| Pathogenic bacteria | | Pathogenic fungi | |
| --- | --- | --- | --- |
| Genus | Ref. | Genus | Ref. |
| *Bacillus* | the Ministry of Health of the People's Republic of China; Black, 2012. | *Coccidioides* | the Ministry of Health of the People's Republic of China; Black, 2012; Ijaz et al., 2016. |
| *Burkholderia* |  | *Histoplasm* |  |
| *Francisella* |  | *Paracoccidioides* |  |
| *Mycobacterium* |  | *Absidia* |  |
| *Yersinia* |  | *Alternaria* |  |
| *Acinetobacter* |  | *Arthrinium* |  |
| *Vibrio* |  | *Aspergillus* |  |
| *Actinobacillus* |  | *Blastomyces* |  |
| *Actinomadura* |  | *Candida* |  |
| *Actinomyces* |  | *Cephalosporium* |  |
| *Aeromonas* |  | *Cladosporium* |  |
| *Afipia* |  | *Cryptococcus* |  |
| *Amycolata* |  | *Dactylaria* |  |
| *Arachnia* |  | *Dermatophilus* |  |
| *Arcanobacterium* |  | *Emmonsia* |  |
| *Brucella* |  | *Epidermophyton* |  |
| *Bacteroides* |  | *Exophiala* |  |
| *Bartonella* |  | *Fonsecaea* |  |
| *Calymmatobacterium* |  | *Fusarium* |  |
| *Campylobacter* |  | *Geotrichum* |  |
| *Clostridium* |  | *Loboa* |  |
|  |  | *Madurella* |  |
| *Corynebacterium* |  | *Microsporum* |  |
| *Dermatophilus* |  | *Mucor* |  |
| *Edwardsiella* |  | *Penicillium* |  |
| *Eikenella* |  | *Pneumocystis* |  |
| *Enterobacter* |  | *Rhizopus* |  |
| *Erysipelothrix* |  | *Sporothrix* |  |
| *Escherichia* |  | *Stachybotrys* |  |
| *Flavobacterium* |  | *Trichoderma* |  |
| *Fluoribacter* |  | *Trichophyton* |  |
| *Francisella* |  | *Trichothecium* |  |
| *Fusobacterium* |  | *Xylohypha* |  |
| *Gardnerella* |  | *Meruliaceae* | Liang et al., 2020. |
| *Haemophilus* |  | *Trametes* |  |
| *Helicobacter* |  | *Periconia* |  |
| *Kingella* |  | *Pleosporales* |  |
| *Klebsiella* |  | *Loweporus* |  |
| *Legionella* |  | *Nigrospora* |  |
| *Listeria* |  | *Leiotrametes* |  |
| *Mima* |  | *Ciliophora* |  |
| *Morganella* |  | *Talaromyces* |  |
| *Mycobacterium* |  | *Stemphylium* |  |
| *Neisseria* |  | *Cercospora* |  |
| *Nocardia* |  | *Ascomycota* |  |
| *Pasteurella* |  | *Cochliobolus* |  |
| *Peptostreptococcus* |  | *Setophaeosphaeria* |  |
| *Plesiomonas* |  | *Chaetomium* |  |
| *Prevotella* |  | *Blakeslea* |  |
| *Proteus* |  | *Curvularia* |  |
| *Providencia* |  | *Choanephora* |  |
| *Pseudomonas* |  | *Malassezia* | Qi et al., 2020; Limon et al., 2017. |
| *Rhodococcus* |  | *Trichosporon* |  |
| *Salmonella* |  | *Scedosporium* |  |
| *Serpulina* |  | *Glomerella* |  |
| *Serratia* |  | *Zasmidium* |  |
| *Shigella* |  | *Pestalotiopsis* |  |
| *Staphylococcus* |  | *Phyllosticta* |  |
| *Streptobacillus* |  | *Epicoccum* |  |
| *Streptococcus* |  | *Selenophoma* |  |
| *Vibrio* |  | *Mycosphaerella* |  |
| *Yersinia* |  | *Sarocladium* |  |
| *Enterococcus* | Fan et al., 2019. | *Schizophyllum* |  |
| *Aerococcus* |  | *Ganoderma* |  |
| *Lactococcus* |  | *Ustilago* |  |
| *Micrococcus* |  |  |  |
| *Stenotrophomonas* |  |  |  |
| *Clostridiaceae_1* | Liang et al., 2020. |  |  |
| *Corynebacterium_1* |  |  |  |
| *Enterobacteriaceae* |  |  |  |
| *Corynebacteriales* |  |  |  |
| *Escherichia-Shigells* |  |  |  |
| *Proteobacteria* |  |  |  |
| *Rhizobiales* |  |  |  |
| *Bacilli* |  |  |  |
| *Burkholderiales* |  |  |  |
| *Burkholderia_Paraburkholderia* |  |  |  |
| *Bacillales* |  |  |  |

**Table S2** Parameters for risk assessment of adults and children

| Parameter | Values | | | Units |
| --- | --- | --- | --- | --- |
|  | adult male | adult female | children |  |
| Inhalation rate (IR) | 17.7 | 14.5 | 7.6 | m^3^·d^-1^ |
| Exposure frequency (EF) | 250 | 250 | 250 | day·year^-1^ |
| Exposure duration (ED) | 25 | 25 | 6 | year |
| Body weight (BW) | 66.1 | 57.8 | 19.6 | kg |
| Average lifetime (AT) | 72.38×365 | 77.37×365 | 12×365 | day |
| Reference dose (RfD) | 1500 | | | CFU·(kg·d)^-1^ |
